# A rapid review of community engagement and informed consent processes for adaptive platform trials and alternative design trials for public health emergencies

**DOI:** 10.12688/wellcomeopenres.19318.1

**Published:** 2023-05-02

**Authors:** Alun Davies, Ilja Ormel, Alexe Bernier, Eli Harriss, Noni Mumba, Nina Gobat, Lisa Schwartz, Phaik Yeong Cheah

**Affiliations:** 1 Health Systems Collaborative, Nuffield Department of Medicine, University of Oxford, Oxford, England, UK; 2 Faculty of Health Sciences, Department of Health Research Methods, Evidence & Impact, McMaster University, Hamilton, Ontario, Canada; 3 Faculty of Social Sciences, School of Social Work, McMaster University, Hamilton, Ontario, Canada; 4 Bodleian Health Care Libraries, University of Oxford, Oxford, England, UK; 5 The KEMRI-Wellcome Trust Research Programme, Kilifi, 80108, Kenya; 6 Nuffield Department of Primary Care Health Sciences, University of Oxford, Oxford, England, UK; 7 Mahidol Oxford Tropical Medicine Research Unit, Faculty of Tropical Medicine, Mahidol University, Salaya, Nakhon Pathom, Thailand; 8 Centre for Tropical Medicine and Global Health, Nuffield Department of Medicine, University of Oxford, Oxford, England, UK

**Keywords:** Adaptive trial, Engagement, Pandemic, COVID-19, Ebola, Informed Consent, Global Health Emergency, Good participatory Practice

## Abstract

**Background *:*** Public Health Emergencies (PHE) demand expeditious research responses to evaluate new or repurposed therapies and prevention strategies. Alternative Design Trials (ADTs) and Adaptive Platform Trials (APTs) have enabled efficient large-scale testing of biomedical interventions during recent PHEs. Design features of these trials may have implications for engagement and/or informed consent processes. We aimed to rapidly review evidence on engagement and informed consent for ADTs and APTs during PHE to consider what (if any) recommendations can inform practice.

**Method *:*** In 2022, we searched 8 prominent databases for relevant peer reviewed publications and guidelines for ADTs/APTs in PHE contexts. Articles were selected based on pre-identified inclusion and exclusion criteria. We reviewed protocols and informed consent documents for a sample of large platform trials and consulted with key informants from ADTs/APT trial teams. Data were extracted and summarised using narrative synthesis.

**Results *:*** Of the 49 articles included, 10 were guidance documents, 14 discussed engagement, 10 discussed informed consent, and 15 discussed both. Included articles addressed ADTs delivered during the West African Ebola epidemic and APTs delivered during COVID-19. PHE clinical research guidance documents highlight the value of ADTs/APTs and the importance of community engagement, but do not provide practice-specific guidance for engagement or informed consent. Engagement and consent practice for ADTs conducted during the West African Ebola epidemic have been well-documented. For COVID-19, engagement and consent practice was described for APTs primarily delivered in high income countries with well-developed health service structures. A key consideration is strong communication of the complexity of trial design in clear, accessible ways.

**Conclusion**: We highlight key considerations for best practice in community engagement and informed consent relevant to ADTs and APTs for PHEs which may helpfully be included in future guidance.

**Protocol**: The review protocol is published online at Prospero on 15/06/2022: registration number CRD42022334170.

## Introduction

Public health emergencies (PHEs) caused by novel or re-emerging pathogens present a significant threat to the health and security of people around the world ^1– 3^. In the wake of the COVID-19 pandemic, preparing for effective responses to these events remains a priority for governments and international agencies such as the World Health Organisation (WHO) ^3, 4^. Clinical research is central to the response ^3, 5^, particularly during novel infectious disease outbreaks where no known medical countermeasures exist ^2^. During a PHE, rapid recruitment of large numbers of research participants are likely to be needed to produce results that can be used during the active phase of an outbreak ^1, 3, 6, 7^. As illustrated in the H1N1 pandemic where no clinical trial evidence was available to inform treatment ^1^, the time needed to establish clinical trials must be optimised to avoid missing outbreak peaks. Learning the lessons from H1N1, COVID-19 saw important advances to rapid production of evidence related to vaccines and therapeutics. These advances reflect innovation to the ways in which clinical research was designed and conducted during PHEs. In particular, the use of alternative design trials (ADTs) and adaptive platform designs (APTs) have advanced the potential for clinical research to produce timely evidence for public health and clinical responses to emergency events ^7– 11^.

Adaptive designs draw from knowledge gained during the trial to inform and amend subsequent actions pre-defined by the study protocol, for example, amendments in doses or target study populations ^12^. APTs are guided by a single master protocol which enables the comparison of several treatments/vaccines against a single control aimed at standardizing study procedures across participating sites and contexts in order to minimize the duplication of efforts, streamline ethical review and achieve adequate, and diverse participation when time is of the essence ^9^. A prominent feature in APTs is the ability to drop arms for futility or harm as trial data emerges, and also to introduce new treatment arms as new products become available for evaluation ^8, 13, 14^. Examples of this type of trial include the PRINCIPLE primary care trial ^15^, TOGETHER ^16^, REMAP-CAP ^17^, RECOVERY ^18^ treatment trials, and SOLIDARITY which used an adaptive platform to evaluate new COVID-19 therapeutics or vaccines in multiple sites and often in multiple countries around the world ^19^. The RECOVERY trial, where recruitment is integrated with healthcare through the UKs National Health Service hospital network, has further been described as a pragmatic APT in that therapies are tested for effectiveness in routine healthcare practice ^20^. ADTs include seamless phase I/II/III trials aim at efficiency through combining RCT phases in a single protocol ^21^, and stepped wedge trials, which may introduce trial arms sequentially. Another ADT is the ring-vaccination cluster-randomised controlled trial design, which was used to randomize rings of individuals in close proximity to index cases for vaccination in West Africa during the 2013-2016 Ebola epidemic ^21^.

Engagement and informed consent are essential elements of clinical trials, important within their own right, but also mutually supportive ^22, 23^. For this review, we use the term engagement to include Good Participatory Practice (GPP) ^24^, Patient and Public Involvement (PPI), consultation, dialogue and information giving/gathering activities, with a range of public, community members and stakeholders, which are used to support, enable and strengthen clinical trials throughout their lifecycle. Guidelines for strong ethical practice for clinical trials, including those conducted during in PHEs, highlight the importance of engagement with communities, patients and publics throughout the conduct of clinical trials and the duty of care that researchers have to ensure that, prior to obtaining signed individual informed consent, participants have been provided with sufficient time and opportunity to consider participation, based on being provided adequate understanding of the research without coercion ^23, 25– 27^. It is currently unclear what, if any, specific considerations are provided for engagement and informed consent processes for ADTs and APTs during PHEs.

This rapid review is part of commissioned project from the WHO Ethics and Governance Unit. It aims to summarise guidance, reports and evidence on implementation of engagement or informed consent practices for ADTs and APTs during PHEs. We describe how challenges raised by the PHE context and the need for expeditious evaluation of new/repurposed therapies and vaccines were addressed. Based on this we make suggestions towards further research and consider implications for guidelines related to ADTs and APTs during PHEs.

## Methods

We aimed to rapidly review evidence on engagement and informed consent for ADTs and APTs during PHE to consider what (if any) recommendations can inform practice by asking the following questions:

1.   What specific considerations, if any, are given to engagement and informed consent for ADTs and APTs during PHEs in prominent guidance documents?

2.   How have stakeholders been engaged for ADTs and APTs during PHEs?

3.   How has informed consent been undertaken for ADTs and APTs during PHEs?

4.   What is the empirical evidence for the success, pitfalls and outcomes of engagement and informed consent approaches reported for ADTs and APTs during PHEs?

As is common for rapid reviews, our approach comprises a systematic literature search, screening and selection of studies, a narrative synthesis and dialogue with knowledge users ^28^. The review protocol is published online at Prospero (published on 15/06/2022): registration number CRD42022334170.

### Eligibility criteria

Articles were included if they met all of the following criteria:

•   Articles documenting or describing stakeholder/public/community/ patient engagement/involvement and/or informed consent to support ADTs and APTs during PHEs – which comprise platform, cluster-randomised ring-vaccination, stepped-wedge, pragmatic, seamless multi-phase and adaptive design trials.

•   Article type: Primary empirical studies, guidelines policy frameworks, programme reports, conceptual/ discussion papers, commentaries and letters to the editor

•   Language: English language.

•   Date: any date

Articles were excluded if they met all of the following criteria:

•   Articles describing engagement and informed consent for non-ADT/APTs (e.g. RCTs), or trials in non-PHE (HIV, TB, Malaria, cancer, asthma, obesity, heart disease etc.), or articles reporting on research during PHEs with no content on engagement or informed consent

•   Article type: Evidence reviews

•   Language: Non-English language.

•   Date: no exclusion based on date

### Information sources and search strategy

We searched the following data bases from their earliest inclusion to July 2022: Ovid Medline, Ovid PsycINFO, Ovid EMBASE, Ovid Global Health, Scopus, Web of Science (All Databases), medRxiv and Google Scholar using a structured search strategy. The search strategy was developed in Ovid Medline and refined for each database (see *Extended data* ^29^). Search terms for four domains were developed based on team experience in the field and drawing on key references ^30^. The domains were linked with Boolean operators as follows:

a) Community engagement and related terms; OR
b) Informed consent and related terms; AND
c) PHEs declared by the WHO (as defined by International Health Regulations ^31^); AND
d) Adaptive platform trials (comprising flexible arm designs and, to diversify the review to include studies conducted in sub-Saharan Africa, stepped-wedge designs, cluster-randomised ring-vaccination designs, and seamless phase I/II and II/III designs.

Supplementary searches were conducted of relevant core journals, snowball searching to check reference lists of included articles, and recommended sources were requested via expert networks. Grey literature was accessed through relevant websites (e.g. WHO, NIHR, RECOVERY trial, REMAP-CAP, PRINCIPLE etc.). Further, we used the following two strategies to further supplement our search on ADTs and APTs during PHEs. The first was a hand search for protocols, participant information sheet and informed consent form for eight prominent adaptive trials for COVID-19. These trials were selected because they are the largest COVID-19 trials in terms of recruitment. They comprise six adaptive platform trials and two large non-adaptive platform trials for comparison. The second strategy comprised holding discussions with engagement and communications leads for two prominent trials (REMAP-CAP and RECOVERY).

### Selection, data extraction and synthesis

Using the criteria above, the principal reviewer (AD) screened titles and/or abstracts of retrieved articles for inclusion/exclusion. A second reviewer (AB) independently screened a sample of 10% to identify any systematic screening errors. Full text articles were subsequently reviewed by the principal reviewer, with 10% independently reviewed by a secondary reviewer (IO). Disagreements between the principal and second reviewers were resolved through re-review and discussion.

The principal reviewer (AD), IO and AB extracted data using pre-defined and piloted data extraction sheets to capture details about the type of article, type of adaptive trial, PHE, engagement and consent approaches, and recommendations. Guidance document identified during the search were grouped together to address question 1. The remaining articles and documents were imported into NVIVO14 ( QDA MINER LITE could be used as an open-access software alternative for our approach) and thematic approach based on the extraction criteria was used to describe the guidance documents and approaches for engagement and informed consent.

## Results

After removing duplicates, database searches (including Google Scholar) and supplementary (back-and-forth citation) searches yielded 469 articles for title and abstract screening. Sixty-four articles were identified, for full text review and from this 15 were rejected because they did not fit the inclusion criteria. Of the 49 documents included in the review, 10 were classified as ‘guidance documents’ because they presented guidance, a framework or related content related to engagement and/or consent to clinical research during PHEs. The remaining 39 comprised 14 articles on engagement, 10 on informed consent, and 15 discussed both concepts within a context of APTs during PHEs. Considerations for community engagement and informed consent for ADTS and APTS during PHES in included guidance documents.

Table 1 summarises ten prominent guidance documents identified in the review. Of these, one gave guidance relevant to Ebola trials ^32^, three for COVID-19 ^33– 35^, four referred to outbreaks or PHEs in general ^2, 3, 23, 27^, and two gave general guidance for engagement in clinical research but not specific to PHEs ^36, 37^. These documents highlight the value of ADTs or APTs and the importance of community engagement but do not provide specific guidance for engagement practice or informed consent for these novel trial designs in PHEs. Of the 10 guidance documents reviewed, 4 gave specific guidance for informed consent ADT/APTs in PHE contexts ^3, 23, 32, 34^.

**Table 1.** Prominent guidance documents relevant to engagement and informed consent for ADTs and APTs during PHEs.

Document	Purpose	Relevance	Engagement		Consent
			Goals	Guidance of relevance to PHE and APTs	Guidance of relevance to PHE and APTs
WHO, (2014) ^32^	Ethical issues related to study design for trials on therapeutics for Ebola Virus Disease: Working Group meeting discussion	PHE context (Ebola) No guidance specific to ADTs or APTs	None explicitly mentioned	• Community input to feasibility and acceptability of trial design.	• informed consent important ethical requirement despite PHE • Capacity strengthening for local research ethics committees regarding review of complex and adaptive study designs • Innovative approaches to aid comprehension and voluntariness. e.g. video or audio recordings or surrogate consent
WHO, (2016) ^27^	Guidance for managing ethical issues in infectious disease outbreaks: WHO (2016)	PHE context (general, infectious disease outbreaks) No guidance specific to ADTs or APTs	Justice, Beneficence, respect for persons, liberty, solidarity, utility, reciprocity	• Community input to appropriateness and acceptability of randomization, placebo controls, blinding or masking and methodology. • Engagement about benefits and risks of monitored emergency use of unregistered and experimental medicines (MEURI) • Engagement during and after the trial important for building trust	• Consent forms and processes to be developed with local communities and local staff • For patients incapable of providing informed consent, proxy consent should be obtained from a family member or other authorized decision-maker
CIOMS, (2016) ^23^	International Ethical Guidelines for Health-related Research Involving Humans	PHE context (general, all disasters) Guidance for cluster randomised trial designs	Respecting communities, Social value, Justice, Ensuring inclusivity and diversity	• Community input to feasibility and cultural sensitivity • Engagement key to build trust, foster community leadership and gain public support for research	• Informed consent important ethical requirement despite PHE: Individual informed consent is obtained even in a situation of duress, unless the conditions for a waiver are met • For persons incapable of giving informed consent, guidelines for deferred/waived consent for all trials apply to APTs in PHE For cluster randomised trials: • Informed consent must be obtained from participants • Where there are community gatekeepers, permission must be gained to enrol the cluster but individual informed consent still required
Hankins, (2016) ^2^	Good participatory practice guidelines for trials of emerging (and re-emerging) pathogens that are likely to cause severe outbreaks in the near future and for which few or no medical countermeasures exist (GPP-EP)	PHE context (infectious disease outbreaks, (re- emerging pathogens) No guidance specific to ADTs or APTs	Respect, Fairness, Integrity, transparency, accountability, Autonomy	• Community input to feasibility, protocol development, trial design and procedures, including the investigational products, trial objectives, recruitment strategies, informed consent, reimbursement policies, counselling approaches, follow-up, and post-trial access. • Engagement to support trial accrual, and follow-up, accounting for socially and culturally appropriate strategies • Engagement for trial exit, using socially and culturally appropriate strategies • Engaging relevant stakeholders about trial closure and transparent dissemination to build trust and lay a positive foundation	No specific guidance given for APTs, but comprehensive guidance given on how Good Participatory Practice and local stakeholder engagement can contribute to informed consent processes including gaining community input on: • Appropriate consenting, including familial or communal consent when patients are incapacitated and children • Appropriate language, literacy level, cultural approaches.
NHS Health Research Authority, (2017) ^36^	UK policy framework for health and social care research	Not specific to PHE No guidance specific to ADTs or APTs	None explicitly mentioned	• Patients, service users and the public are involved in the design, planning, management, conduct and dissemination of research, unless otherwise justified.	No guidance given for informed consent for APT in PHE contexts
NIHR, (2019) ^37^	UK Standards for Public Involvement in Research: Better public involvement for better health and social care research	Not specific to PHE No guidance specific to ADTs or APTs	None explicitly mentioned	• Early involvement important • Public should be involved in decision- making • Public should be involved in documenting, assessing the impact of PPI, and reflecting on the findings	No guidance given for informed consent for APT in PHE contexts
WHO, (2020) ^35^	Good Participatory Practice for COVID-19 clinical trials: a toolbox – based on GPP-EP	PHE context (COVID-19) No guidance specific to ADTs or APTs	None explicitly mentioned	• Community input to feasibility and acceptability of delivering the trial, including feedback about their contexts language, translation needs and community entry points • Engagement to monitor feedback and be responsive to concerns, tensions rumours • Evaluate and share lessons learned • Engagement for trial exit, using socially and culturally appropriate strategies	Community consultation should inform appropriate consent
The Nuffield Council on Bioethics working group, (2020) ^3^	Research in global health emergencies: ethical issues	PHE context (general, global health emergencies) Limited guidance on ADTs or APTs	Building mutually respectful partnerships, Aspiring to achieve joint ownership, Creating well- founded trust in research	• Community input to feasibility and acceptability of study protocols • Even in multi-site trials, there will be elements that can and should be operationalised differently in different sites in response to engagement and feedback.	Challenges in understanding complex trial designs, and difference between care and research (therapeutic misconceptions), heightened by emergency contexts (disruption, family separation, lack of access to basic resources and services, fear, distress, powerlessness, lack of alternative options, difficulties in communication, unequal power dynamics, lack of protective equipment):, the wider ethic ‘ecosystem’ can be drawn upon to strengthen and support ethical practice, for example stakeholder and ethics review committee engagement towards agreeing on ethical procedures. When reviewing proposed consent processes, research ethics committees should consider: • if proposed consent processes are the best and most sensitive possible in the circumstances; • other requirements to ensure respect for participants as people of equal moral worth and agency; and • whether, in all the circumstances, what is being asked of participants can be justified as fair.
NIHR, (2021) ^33^	Briefing notes for researchers - public involvement in NHS, health and social care research:	Not specific to PHE No guidance specific to ADTs or APTs	Intrinsic to citizenship, public accountability and transparency, mutual respect	PPI necessary for: • Identifying and prioritising research questions • Shaping research methods and commenting on the feasibility • Contributing to grant applications • Giving input into information sheets and other documents • Undertaking research projects • Contributing to how study results are disseminated, either by advising researchers, presenting at conferences, or via social media.	Collaboration with PPI groups can help with recruitment and informed consent
Singh, (2020) ^34^	WHO guidance on COVID-19 vaccine trial designs in the context of authorized COVID-19 vaccines and expanding global access: Ethical considerations	PHE context (COVID-19) Guidance for placebo control vaccine trials including adaptive designs	None explicitly mentioned	• Stakeholder and community input to trial feasibility and acceptability	For placebo controlled trials, at informed consent, participants must be informed that they can be unblinded where efficacious vaccines become available.

### Community engagement

Guidance documents highlight constraints to engagement in the context of PHE, including the urgency for action, the extent to which lay members of the public can inform complex biomedical research ^3^, and the extent to which local communities can influence decisions on multi-country standardised master-protocols of the sort used in platform trials during PHE contexts ^35^. Despite these constraints, researchers have a moral duty to deliver community and stakeholder engagement throughout the lifecycle of clinical trial ^3, 27, 33^. Prominent aims of community engagement include understanding community norms, values and traditions ^3, 27, 35^; identifying research priorities/questions ^23, 33, 35^ informing trial designs ^2, 23, 33, 35, 36^; assessing acceptability ^3, 23, 27, 32^ and feasibility ^27, 33^ of trial designs; listening and respond to community concerns ^23^; and shaping public facing trial documents such as informed consent forms ^2, 33, 35^.

Guidance documents highlight the importance of documenting communications or engagement plans ^2, 23, 33, 35, 37^, and including these in funding applications ^3, 33^ and trial protocols ^2, 23, 33^. Community Advisory Boards and/or Patient and Public Involvement (PPI) groups are recommended for gaining diverse perspectives from affected communities and informing trial implementation ^2, 3, 35, 38^. Practical guidance on how to work with CABs/ PPI groups included regarding working online with these groups during social distancing measures ^33, 38^. No guidance is given specifically on engagement with CABs and PPI groups for APTs during PHEs.

Throughout the implementation of clinical trials, documenting, monitoring and evaluating engagement activities and outcomes is recommended ^23, 35, 36^. Active CAB engagement can help to monitor community tensions, views and rumours, and advise on how to appropriately respond to them through contributing to culturally sensitive communications ^2, 33, 35^. This engagement is aimed at ensuring the trial is responsive to community views ^36^ and support study accrual and follow-up ^2^. Little guidance is offered on engagement specific to the closure of adaptive platform trials in PHE settings. For clinical trials in general, several guidelines recommend stakeholder/community involvement in dissemination of results ^2, 23, 33, 35, 36^, and in including stakeholders in the planning and implementation of exit strategies ^2, 35^.

### Informed consent

Guidance provided includes need for Ethics Review Committee capacity strengthening ^3, 32^, for innovative approaches (for example using audio-visual aids) to be used to support individual informed consent ^32^, for individual as well as ‘community gatekeeper’ (e.g. in West African countries) consent for ring vaccination cluster randomised trials ^23^. For vaccine trials informed consent should inform participants that they can be unblinded when efficacious vaccines become available ^34^. Consent processes and materials should be developed in collaboration with community stakeholders ^2, 3, 27, 35^. Guidance is also given on deferred, waived consent and consent by proxy in PHE contexts for all trials ^2, 3, 23, 27^. The following constraints to informed consent were highlighted: understanding complex (adaptive) trial designs ^3, 32^, differentiating between care and research ^3, 27^, and challenges raised by the PHE context, for example, fear, lack of alternative options, disruption, family separation, lack of access to basic resources and services ^3, 23, 27^.

### Community engagement for ADTs and APTs during PHEs

Of the 29 included articles, 22 were discussion papers reporting expert views, opinions or experiences, 5 were reports of primary empirical research using qualitative (focus groups and interviews) and quantitative methods (public survey), and 2 were trial reports with no empirical data on engagement ( Table 2). The majority of papers considered clinical research conducted during the Ebola epidemic in West Africa (16 of 29) and COVID-19 (9 of 29). The term “community engagement” was used most widely (19 of 29), and “social mobilisation” was also used ^39– 41^. Public engagement and/or involvement was used for COVID-19 practice ^14, 42, 43^, and UK-based COVID-19 trials used Patient and Public Involvement (PPI) ^33, 43^. One article spoke of Good Participatory Practices (GPP) ^44^.

**Table 2.** Characteristics of included articles describing community engagement for ADTs and APTs during PHEs.

	Citation	Type of article	Main focus of article	PHE	APT/ADT	Key finding on content relevant to engagement	Objective of engagement and approach
Discussion papers							
1.	Alirol *et al*., (2017)	Discussion	Report on the experience of WHO Ethics Review Committee regarding Ebola clinical trials	Ebola 2014–2016 West Africa (Guinea)	Ebola Ca Suffit Ring vaccination cluster randomised trial Guinea	Protocols submitted had insufficient descriptions of community engagement and ERCs were concerned about: (1) understanding of risks related to receipt of immediate vs delayed vaccine; (2) exclusion of pregnant women and children who were at highest risk.	• To support understanding of risk • To maximise understanding of measures to reduce transmission • To support trust in research and facilitate implementation • Close collaboration • of local and international researchers
2.	Browne *et al*., (2018)	Discussion	Strategies used to retain participants in clinical research	Ebola 2014–2016 West Africa (Liberia)	PREVAIL Ebola vaccine trial	Recruited local trackers for community mobilisation and follow-up and used illustrated flipbooks to support the understanding trial information. Several community meetings were facilitated	• Support trial retention • Raise awareness, respond to community questions, concerns and rumours
3.	Folayan *et al*., (2016)	Discussion	Addressing ethical concerns in the development of therapies for Ebola infection management and its prevention	Ebola 2014–2016 West Africa	Ebola Vaccine trials in West Africa	Recommends: • Broad stakeholder engagement at local, regional and international levels using available communication channels • Healthworker engagement and their access to trial participation should be prioritised • Culturally sensitive engagement needed	• Ensuring research plans are relevant and acceptable • Support trial participation • To support long-term research literacy and address rumours, myths, therapeutic misconception and stigma • Promoting local ownership
4.	Goossens *et al*., (2021)	Discussion	Argues for the creation of structures and partnerships to facilitate clinical research, and simplification of clinical trial delivery	COVID-19	REMAP-CAP and SOLIDARITY treatment trials	Large APTs were facilitated rapidly through coordinated high-level engagement and master protocol with no room for local input	High level stakeholder description described towards facilitating APTs across hospitals in the UK
5.	Henao- Restrepo *et al*., (2015)	Discussion	Describing trial implementation	Ebola 2014–2016 West Africa (Guinea)	Ebola Ca Suffit Ring vaccination cluster randomised trial Guinea	• Community resistance to the trial was encountered • Engaging community leaders played a key role in the recruitment strategy	• Seeking community permission Ring identification and recruitment
6.	Higgs *et al*., (2017)	Discussion	Describing trial implementation	Ebola 2014–2016 West Africa	EBOV, PREVAIL, STRIVE and Ebola ca Suffit trials in West Africa	Thoughtful and intensive community engagement in each country enabled the critical community partnership and acceptance of the phase II/III in each country	• To gain community perspectives on study designs, promote acceptance, address fears and misconceptions, and provide social support for enrolled community members to support recruitment and retention
7.	Lane *et al*., (2016)	Discussion	The conduct of clinical trials in outbreak settings	Ebola 2014–2016 West Africa	PREVAIL trial in Liberia	Forming strong local partnerships is resource and time-intensive but essential for conducting ethically and scientifically sound trials	• Avoid exploitation and respect for volunteers
8.	Larson *et al*., (2017)	Discussion	The conduct of clinical trials for Ebola	Ebola 2014–2016 West Africa (Liberia)	PREVAIL trial in Liberia	Intensive engagement using a range of ways and with stakeholders at different levels (community, local leaders, to the vice president) were used to gain public support for the trial	• Participant accrual • Gaining community support for the trial
9.	The UPMC REMAP-COVID Group, on behalf of the REMAP-CAP Investigators, (2021)	Discussion	Description of the REMAP-COVID trial implementation	COVID-19	REMAP-COVID treatment trial	• Engaging with various hospital leaders was essential for operationalising the trial and drawing on the resources of the hospital • No primary data on engagement presented	• Operationalising the trial within hospitals
10.	McMillan *et al*., (2021)	Discussion	Ethical analysis of APTs	COVID-19	Adaptive trials in general	• Trials guided by master protocol to ensure uniformity of procedures across sites • Article highlights the importance of community engaged research and stakeholder involvement at the beginning • No primary data on engagement presented	• To address ethical principles of autonomy, beneficence and justice • To support autonomy through strengthening health literacy
11.	National Academies of Sciences, Engineering, and Medicine (2017)	Discussion	Expert deliberation about trial implementation	Ebola 2014–2016 West Africa (Guinea, Liberia and Sierra Leone)	A range of Ebola trials in West Africa	• Describes historical basis for mistrust in research and inadequate engagement at the outset resulted in resistance to research • Subsequent wider engagement and consultation facilitated a greater degree of mutual understanding and acceptance • Stakeholder deliberation was conducted towards agreement on trial design • National-level agreements with international researchers for the conduct of clinical trials during an epidemic do not necessarily indicate local acceptance • International response and research teams would be strengthened by the inclusion of social scientists with expertise in community engagement	A comprehensive range of goals for engagement given including: • To listen to and respect community views • Building community knowledge and capabilities about the emergency • Incorporating community priorities and perspectives into epidemic response and research plans • Address rumours and fears • Building community trust and confidence in researchers, • Recruitment in clinical trials
12.	Papadimos *et al*., (2018)	Discussion	A review of the ethics of research in GHE and expert consensus	General Global Health Emergencies	Trials (including APTs) during outbreaks drawing on West Africa experience	None	• To inform decision-making on the allocation of resources
13.	Park *et al*., (2021)	Discussion	Discussion paper arguing the case for APTs - how COVID-19 has changed clinical research in global health	COVID-19	Adaptive trials in general	No primary data on engagement presented	• To formulate important research questions based on community health priorities • To improve contextual understanding of the study region so that appropriate interventions and trial design strategies are developed
14.	Patel *et al*., (2021)	Discussion	Description of engagement tools and approaches	COVID-19	PRINCIPLE evolving arms treatment trial	No primary data on engagement presented	Raise awareness among BAME communities and support inclusion of diverse groups into the trial
15.	Ratneswaren *et al*., (2020)	Discussion	Community and patient involvement in COVID-19 research	COVID-19	Adaptive trials in general	Argues that gathering community views can be done in time constrained and emergency contexts	• To include public views into health research • To support recruitment
16.	Salerno *et al*., (2016)	Discussion	Discusses a plenary session about the ethics of trials in pandemics	Ebola 2014–2016 West Africa	Ebola trials (including ADTs) in West Africa	• No primary data on engagement presented	• Gathering community support • Maintaining trust, addressing rumours and concerns, reporting to the community, respecting community diversity, prevention of spread
17.	Saxena and Gomes, (2016)	Discussion	Ethical challenges during the Ebola outbreak	Ebola 2014–2016 West Africa	Ebola trials (including ADTs) in West Africa	No primary data on engagement presented • At the outset there was limited initial engagement, panic, fear, lack or credible information and rumour • Community-based research important for Guinean ring vaccination trial • The degree of engagement required and the ‘‘community’’ to be engaged depends upon the intervention envisaged (e.g. hospital based for treatments versus community based vaccines). • Criteria are lacking to assess whether a particular strategy for community engagement is adequate. • Most protocols mention some CE but it is not always described in articles	Providing community input into trials
18.	Saxena, (2014)	Discussion	The ethics of health system responses to the Ebola outbreak	Ebola 2014–2016 West Africa	Ebola trials (including ADTs) in West Africa	• No primary data on engagement presented	• Being transparent with community members • To support trials
19.	Thielman *et al*., (2016)	Discussion	Lessons learned for the implementation of clinical trials during an outbreak	Ebola 2014–2016 West Africa	Ebola trials (including ADTs) in West Africa	• No primary data on engagement presented • Engagement recommended	To satisfy the necessary administrative, ethical, and regulatory procedures required for research
20.	Thompson, (2016).	Discussion	The ethics of trials during outbreak contexts	Ebola 2014–2016 West Africa	Ebola trials (including ADTs) in West Africa	• No primary data on engagement presented • What exactly is entailed by community engagement in research within the context of a public health emergency needs further work, as well as how to do it ethically and meaningfully so that public trust is built and maintained	• Ensuring acceptability of trials • Building and maintaining public trust
21.	Tikkinen *et al*., (2020)	Discussion	Implementation of SOLIDARITY and RECOVERY COVID-19 trials	COVID-19	RECOVERY and SOLIDARITY treatment trials	• No primary data on engagement presented	Engaging high-level stakeholders and hospital manages to operationalise the trials
22.	Wilson *et al*., (2021)	Discussion	Good Participatory Practice for research in pandemics	General Global Health Emergencies - Ebola 2014–2016 West Africa and COVID-19	ADT/APTs in epidemics including for Ebola and COVID-19	• Article supports the use of GPP for clinical trials • No primary data on engagement presented	• Allowing stakeholders to contribute towards achieving the trial goals • Form collaborative partnerships • Build trust and address mistrust and rumours
Empirical							
23.	Dimairo *et al*., (2015)	Empirical – qualitative	Qualitative research involving UK-based health and research stakeholders	Ebola 2014–2016 West Africa and influenza scenarios	Adaptive trials in general	• Stakeholders were supportive of adaptive designs but some were unfamiliar • Education recommended to raise stakeholders understanding of APTs	To understand health and research stakeholders understanding and views about adaptive trials
24.	Nichol *et al*., (2021)	Empirical – qualitative study of healthcare/ humanitarian workers	Exploration of participant views on adaptive trials, engagement and consent	Ebola 2014–2016 West Africa	Ebola trials (including adaptive trials) in West Africa	• All participants supported adaptive trials but highlighted the need to engage and involve communities to make ensure acceptability • Guidelines for CE provided	• Promote collaboration • Incorporate community insights into decision-making • reflect cultural values and norms • encourage transparency • foster trust and relationships • and address rumours and fears
25.	Cake *et al*., (2022)	Empirical – quantitative (public survey)	Survey exploring public views about trial recruitment strategies	COVID-19	PRINCIPLE	• Survey respondents were generally supportive of use of test records for telephone recruitment • The approach increased recruitment	To develop and appraise the use of NHS records and a text- messaging service to recruit/ follow-up participants
26.	Gobat *et al*., (2019)	Empirical – quantitative (public survey)	To understand public views regarding participation in clinical research during a hypothetical influenza pandemic.	Influenza-like pandemic scenario	• Hypothetical APTs • Therapeutics – primary care and intensive care scenarios • Response adaptive randomisation	• The study concludes strong support for participation in adaptive trials for therapeutics, primary care and intensive care research during pandemic scenarios • Tailored information and initiatives to advance research literacy and maintain trust are required to support pandemic-relevant research participation and engagement.	Public survey (n=6804) in Belgium, Poland, Spain, Ireland, UK, Canada, Australia and New Zealand to understand public views regarding participation in clinical research during a hypothetical influenza pandemic – to inform outbreak response for clinical studies.
27.	Gobat *et al*., (2018)	Empirical –qualitative - focus groups and interviews	To identify public views regarding provision of information and consent to participate in primary and critical care clinical research during a future influenza- like pandemic (Belgium, Spain, Poland and the UK)	Influenza-like pandemic scenario	• Hypothetical APTs • Therapeutics – primary care and intensive care scenarios • Response adaptive randomisation	• Participants were supportive of APTs and for more proportionate research protection procedures for publicly funded, low- risk studies. • Participants prioritised information regarding participation risk over detailed study descriptions within consent information • Public health information distributed through official channels was seen as trustworthy • Public engagement necessary to address therapeutic misconception	To identify public views regarding provision of information and consent to participate in primary and critical care clinical research during a future influenza- like illness pandemic
Publications – trial reports							
28.	Henao- Restrepo *et al*., (2017)	Publication – Trial report	Describing trial implementation – no empirical data on engagement	Ebola 2014–2016 West Africa (Guinea and Sierra Leone)	Ebola Ca Suffit Ring vaccination cluster randomised trial Guinea	Close collaboration and support at National level contributed to successful trial implementation of the trial.	Obtain community consent to ensure full ownership and understanding by national authorities and communities
29.	Sholzberg *et al*., (2021)	Publication – Trial report	Trial report– no empirical data on engagement	COVID-19	Randomised controlled, adaptive, open label clinical trial.	• No primary data on engagement presented • Authors specify that no engagement was done	None mentioned

### Ebola 2014-2016 ADTs

For trials in West Africa during the 2014-2016 Ebola epidemic, ADTs included cluster randomised ring vaccination and seamless phase I/II/III designs. Health and research stakeholders debated the ethical acceptability of randomisation and use of placebo in RCTs given the high mortality of Ebola disease ^45– 47^ and ADTs such as the cluster-randomised ring-vaccination trials were considered more acceptable ^46, 47^. Against a backdrop of public distrust of research and researchers ^39, 44, 45, 48^, early engagement in West Africa reportedly promoted acceptability and buy-in for trial participation ^40, 45^, and the incorporation of community views into decision-making about trial design and implementation ^45^. Early community engagement was described as important for informing respectful entry into the community ^40, 45^, providing an understanding of traditional practices in disease transmission and changing attitudes ^2, 3, 45^, and for appreciating that community health priorities did not always match health challenges explored in trials ^44, 49^. Early engagement also informed social and cultural sensitivity ^40^, for example, in guiding the need for individual and community consent ^46^, and through guiding trial-related consenting, communication and messaging ^39, 40, 44, 45^.

Community engagement aimed at raising awareness and understanding of trials was described as being key to strengthening relationships, trust and support for trials ^40, 44, 46, 50– 52^. Explaining the purpose and procedures of research and providing accurate information was considered important to address rumours and misconceptions ^40, 45, 47, 49, 51^ which could cause stigma and fear ^39^ and consequently slow down recruitment ^44, 49^. Rumours were described as being based on a lack of accurate information on Ebola and research in the community ^45, 47^ and, in settings where research was led by foreign researchers, mistrust, based on a history of colonial oppression ^44^. In Liberia, for example, research participation was likened to guinea-pig experimentation ^45^. Trial information was shared with communities through community meetings and question and answer sessions ^39, 40, 45^, engaging leaders including traditional healers ^39, 40, 44– 46, 50, 51^ and working with nurses to relay messages ^39^, and through distributing information materials ^40^. Engagement with community members also enabled researchers to select appropriate sites for research clinics to minimise stigma ^45^ and undertake disease surveillance and trace contacts ^40^. Engagement strategies which were reported to strengthen retention of trial participants included ensuring that feedback from the community on rumours informed community engagement messages, that messages were given by hospital nurses, the use of local dialects was used and employing local ‘trackers’ to follow-up with trial participants ^39^.

### COVID-19 APTs

For COVID-19, included articles primarily address APTs delivered in high income countries with well-developed health services and structures ( Table 3). A publication of the RAPID trial explicitly stated that no PPI was conducted because of funding limitations and COVID-19 restrictions ^53^. However, other reports and personal communication with trial personnel illustrate multi-stakeholder engagement in APTs ^6, 10, 14, 43, 54– 56^. In its trial protocol, PRINCIPLE (Platform Randomised Trial of Treatments in the Community for Epidemic and Pandemic Illnesses), a UK national primary care platform trial for investigating repurposed therapies for COVID-19 treatment of older people in the community at high risk of complications, describes that prior to implementation, a panel of seven members of the public from the target recruitment age were offered an opportunity to review trial outcomes and design ^15^. The trial team report innovative approaches to inclusive recruitment among socioeconomically deprived and minority ethnic communities. These efforts included targeted efforts to reach diverse audiences in many languages through local and UK national media channels ^55^. They also conducted an online public survey to assess public acceptability of using contact data derived from COVID-19 tests to recruit participants ^42^.

**Table 3.** COVID-19 platform trial protocol and informed consent review.

Trial	Protocol description of engagement/ involvement	Consent approach	Information in ICF related to adaptive trial features
**TOGETHER:** A multi-center, adaptive, randomized, platform trial to evaluate the effect of repurposed medicines in outpatients with early coronavirus disease 2019 (COVID-19) and high- risk for complications: the TOGETHER master trial protocol ^16^	No description given	• Face-to-face signed consent required	• The 5 drugs (in each arm) are named in the consent and the risks associated with each drug stated • The ICF describes procedures to participants for when the trial is "interrupted" by the researcher • "If the study is interrupted you will be notified and the study doctor will take all the measures for your treatment to be continued by the attending team (your referral) in order to continue your treatment with no harm to you"
**The UPMC OPTIMISE-C19** (OPtimizing Treatment and Impact of Monoclonal antIbodieS through Evaluation for COVID-19) trial: a structured summary of a study protocol for an open-label, pragmatic, comparative effectiveness platform trial with response-adaptive randomization ^63^	No description given	• Verbal (described in the protocol - ICF not publicly available)	• UPMC requires physicians to review with patients the fact Sheet for each of the randomisations mAB (monoclonal antibodes) arms, and to discuss the risks and benefits of mABs with patients, with the option to receive a mAB as part of routine care, should they desire mAB treatment. • Patients are told which mAB they are receiving, and physicians and patients can agree to the assigned mAB or request a specific mAB.
**PRINCIPLE:** Platform Randomised trial of INterventions against COVID-19 In older peoPLE: protocol for a randomised, controlled, open-label, adaptive platform, trial of community treatment of COVID-19 syndromic illness in people at higher risk ^15^	• Five women and two men in the target recruitment age group reviewed patient- facing materials, patient information sheet and daily diary, suggesting edits for clarity. They also reviewed outcomes, and trial delivery plans • Trial Steering Committee (with 2 members of the public) reviewed patient-facing materials and commented on study design and dissemination.	• Online consent, with patient information leaflets, pictorial aids and the opportunity to call trial staff for more information. • Phone consent also possible	• Participants informed that procedures may differ for some arms and that they will be contacted to discuss this where appropriate • No other relevant details about the adaptive nature of the trial is provided in the consent form
**REMAP-CAP:** Randomized, embedded, multifactorial adaptive platform trial for community-acquired pneumonia 2020. ^17^	No description given	• Participants were given the consent form and time to read and then a time was arranged to discuss with the researchers by phone. • Participants would then send the signed form. • Approval for ‘deferred’ consent granted in 13 countries	Assent information form contains • Description of all therapeutic arms including side effects for each • Informs relatives of patients that “The study looks at its results as it goes and uses the results so that new patients in the study have a better chance of getting better treatments.
**SOLIDARITY** (therapeutics): An international randomised trial of additional treatments for COVID-19 in hospitalised patients who are all receiving the local standard of care ^64^.	No local input: *"When local ethics committees* * review this international protocol, it can be* * approved (after which the study can proceed* * at that locality) or rejected (in which case it will * *not proceed) but cannot be altered. Likewise,* * any substantial amendments made centrally to * *the core protocol or consent procedure while* * the trial is in progress can only be approved or* * rejected by local ethics committees."*	• Written consent required • Allowance for non-face-to-face methods where social restrictions are a barrier • Deferred consent allowed only when. Authorised by local IRB. Consent given by patient representative and witnessed by proxy	• Information given about the three treatment arm drugs and their possible side-effects and risks • No other information given in relation to the adaptive nature of the trial
**RECOVERY:** Randomised evaluation of COVID-19 therapy ^65^.	No description given	• Informed consent obtained from each patient prior to enrolment • Patients who lack capacity to consent due to severe disease, and for whom a relative to act as the legally designated representative is not available (in person), randomisation and consequent treatment will proceed with consent provided by a clinician (independent of the trial) (if allowed by local regulations). • If they regain capacity, such participants should be provided with information about the trial (ideally prior to discharge, but otherwise as soon as possible thereafter), what their rights are and how to exercise them, but it is not necessary to obtain their written consent. • For children aged <16 years old consent will be sought from their parents or legal guardian.	• ICF updated with trial findings (effectiveness of dexamethasone) • Information given about all treatment arm drugs and their possible side-effects and risks • No other information given in relation to the adaptive nature of the trial
**PANORAMIC:** Platform Adaptive trial of NOvel antiviRals for eArly treatMent of COVID-19 In the Community ^66^.	No description given	Either face-to-face, or by telephone.	Non-adaptive platform trial
**COVID-OUT:** Early Outpatient Treatment for SARS-CoV-2 Infection (COVID-19) ^67^.	No description given	Options for consent comprise: • Self-consent online • Traditional conversation consent • Consent over the phone	Non-adaptive platform trial

High level health and political stakeholder discussions were described as key to the successful and rapid implementation of the RECOVERY, SOLIDARITY, REMAP-CAP and UPMC Remap-Covid COVID-19 therapeutic trials ^6, 43, 44, 54, 56^. Given the lack of validated therapies and the urgent need for research at the early stages of the COVID-19 pandemic, clinicians were engaged to facilitate the RECOVERY and SOLIDARITY trials and to have clinical interventions to offer very sick patients guided by protocol ^6, 57^. An internal report by the RECOVERY’s communication team describes consultation with the department’s pre-existing Public Advisory Panel about the trial, and the subsequent establishment of a RECOVERY-specific panel ^58^. REMAP-CAP and RECOVERY clinical trial teams described regular and extensive communications and interviews with local and mainstream media, where trial principal investigators were often directly involved. PPI groups and advisory panels also contributed to reviewing consent forms and animations, drafting communications to trial participants and advocating for trial participation through several media initiatives (LM Hayes (REMAP-CAP investigator), personal communication, 10 ^th^ May 2022; and A Whitehouse (RECOVERY communications lead), personal communication, 18 ^th^ June 2022).

### Acceptability of ADTs and APTs

Empirical studies have highlighted general acceptability of ADTs and APTs among key stakeholder groups. A qualitative study among international health and research staff found support for adaptive/alternative designs and highlighted the importance of trial-specific early engagement to promote collaboration, incorporating community insights into decision-making, reflecting cultural values and norms, encouraging transparency, fostering trust and relationships and addressing rumours and fears ^59^.

Two empirical articles considered a hypothetical Influenza-like pandemic scenario to inform clinical research preparedness for the Platform for European Preparedness Against Re-emerging Epidemics (PREPARE). PREPARE hosted an EU-wide primary care network as well the REMAP-CAP network. These studies were conducted to inform clinical research preparedness regarding public acceptability of APT designs and alternate models of consent that may be better suited to a pandemic context. These studies identified broad support for APTs ^60, 61^. Publics generally supported the need for research during pandemics, a moderate proportion expressed willingness to participate, and publics shared their preferences on different approaches to informed consent ^60^. Regarding adaptive designs, participants considered it less important to understand the scientific design considerations and more important to know what the implications of the adaptive designs on their participation, including on risks, benefits and burdens ^61^. Qualitative approaches offered members of the public and health stakeholders a greater opportunity to share their views on adaptive trials ^61, 62^. Whilst a few participants felt that dropping or introducing new trial arms may convey uncertainty on the part of researchers, members of the public generally supported the need for more streamlined enrolment procedures, consent waivers for very low risk studies, and the use of routinely collected medical data for research purposes ^61^. Dimairo *et al*., in their qualitative study report clear support for adaptive trials from UK health and research stakeholders but that educative sessions would be required to address general unfamiliarity with adaptive methods ^62^.

### Informed consent for ADTs and APTs during PHEs

Of the 25 included articles, 17 were discussion papers reporting expert views, opinions or experiences, 4 were reports of primary empirical research using qualitative (focus groups and interviews) and quantitative methods (public survey), and 4 were trial reports with no empirical data on engagement ( Table 4). The majority of papers considered clinical research conducted during the Ebola epidemic in West Africa (10 of 25) and COVID-19 (12 of 25).

**Table 4.** Characteristics of included articles describing informed consent for APTs during PHEs.

	Citation	Type of Article	Main focus of article	PHE	ADT/APT	Informed consent approaches and findings	Recommendations
	**Discussion**						
1.	Alirol *et al*., (2017)	Discussion	Report on the experience of WHO Ethics Review Committee regarding Ebola clinical trials	Ebola 2014–2016 West Africa (Guinea)	Ebola Ca Suffit Ring vaccination cluster randomised trial Guinea	• Participants likely to be sick, isolated, aware of their risk of death and likely to perceive participation as their only chance for survival. • Consent done by staff wearing protective equipment with limited discussion time • For two protocols, consent to retrieve anonymized information from patient records was waived because data would improve understanding of the disease (high social value) and seeking consent from previous, sometimes deceased patients, would have been impractical.	• An ERC could modify/ waive informed consent requirements when truly informed voluntary consent is unlikely. • ERC mindful of challenges, suggested ways of easing information and consent procedures through simplifying and reducing information, • Encouraging dialogue while EVD diagnosis was being confirmed and before isolation were proposed to increase understanding and reduce the risk of ‘situational coercion’.
2.	Almufleh and Joseph, (2021)	Discussion	The role of pragmatic clinical trials in guiding response to global pandemics	COVID-19	APTs in general but refers to REMAP-CAP	Short recommendation on consent processes in general	Recommends streamlining "lengthy" and inefficient consent processes for pragmatic trials embedded into care and remote consent processes.
3.	Bierer *et al*., (2020)	Discussion	Ethical challenges in clinical research during the COVID-19 pandemic	COVID-19	Clinical trials including APTs	Consent processes during PHE, including remote e-consent processes	The article questions the validity of e-consent where participant identity cannot be verified and recommends that meeting the urgent demand for new treatments and vaccines in PHE contexts must be balanced by clinician's obligation to get quality informed consent
4.	Browne *et al*., (2018)	Discussion	Anecdotal review of participant retention strategies used during the PREVAIL I vaccine trial.	Ebola 2014–2016 West Africa (Liberia)	PREVAIL I - Phase II/III Trial	Successful consent practice included: • Preparing high quality consent materials and illustrated flip-books • Community group information sessions followed by individual consent	Comprehensive, culturally sensitive, visual informed consent recommended
5.	Casey *et al*., (2022)	Discussion	Comparison of explanatory RCT with pragmatic platform adaptive design for COVID-19 therapeutic trial	COVID-19	RECOVERY	None	• Alteration or waiver of consent for trials comparing therapies that patients would receive as part of routine care • Concern raised about the quality of informed consent administered by clinicians with a ‘lack of training’ – • Community consultation, public disclosures and family/patient notification recommended to express respect for persons
6.	Henao- Restrepo, (2015)	Discussion	Description of the Ebola Ca Suffit trial implementation – methodological paper	Ebola 2014–2016 West Africa (Guinea)	Ebola Ca Suffit! Guinea ring vaccination trial	• Consent sought form community members within the ring • Community leaders engaged • Consent sought prior to randomisation to avoid selection bias	None
7.	The UPMC REMAP-COVID Group, on behalf of the REMAP-CAP Investigators, (2021)	Discussion	Description of the REMAP-COVID trial implementation - methodological paper	COVID-19	REMAP-COVID	• A successful hybrid face to face and ‘teleconferencing’ consenting is described • Technological challenges experienced with remote consenting (for example, internet bandwidth) and patient non-familiarity with technology	• Set up mock-enrolments to test remote process • Engage bedside providers to assist patients as needed • Leverage institutional videoconferencing tools • Intermittent competency training of research personnel • For telephone consenting, video call option should be made available if desired by the patient • If face-to-face consent is allowed, maintaining a physician investigator call pool is recommended to facilitate discussion with a physician
8.	Larson *et al*., (2017)	Discussion	Description of the PREVAIL trial implementation	Ebola 2014–2016 West Africa (Liberia)	PREVAIL – Phase II-III cluster randomised ring vaccination trial	• Illustrative storyboards used to address language and comprehension challenges in informed consent • Audio-visuals attempted but dropped because of undependable power and the need to re-film amended consent procedures	None
9.	Monach and Branch- Elliman, (2021)	Discussion	Waiver of informed consent for minimal risk trials	COVID-19	APTs in general	Varying levels of consent, including waiver (opt- out), oral consent (opt-in) consent) and written consent described for a different range of trials and type of trial candidate, ranging from RCTs for different doses of drugs with known safety profiles, off-label safe drugs and investigational drugs	Trial designs with clinical equipoise that mimic clinical decision-making in which no data are generated outside of usual care which confer minimal additional risk, can be conducted with minimal documentation of consent, even when interventions contain different risks.
10.	Murray *et al*., (2021)	Discussion	Design and implementation of an international, multi- arm, multi-stage platform master protocol covid-19 antiviral agent trial	COVID-19	TICO/ACTIV-3	Modular information sheet with additional information sheets on individual drugs, and their side-effect profile and consent form, minimizes duplication for regulatory and site staff	None
11.	National Academies of Sciences, Engineering, and Medicine, (2017)	Discussion	Expert deliberation about trial implementation	Ebola 2014–2016 West Africa (Guinea, Liberia and Sierra Leone)	A range of Ebola trials in West Africa	• At the time of consenting participants may be ill, fearful, hopeful, expectant, vulnerable and/or confused • Shortened consent forms were used because of challenging context (described above) • Concerns raised that foreign care providers potentially perceived as providing lifesaving treatments, could enhance therapeutic misperceptions • For the Ebola Ca Suffit trial, rings were randomly allocated before individual informed consent is obtained, but participants were informed of the allocation after the consent process to prevent allocation bias • To support consenting the STRIVE trial facilitated 175 information sessions and a hotline for Q&A • Community and individual consent were acquired • EBOVAC-Salone used illustrated flipcharts • Guinea ring vaccination trial used literate witnesses to help explain • Waiver of consent approved in emergency situations, but opt out mechanisms were used for people who did not wish to participate in the form of wristbands or bracelets • Consent by proxy deemed appropriate where patients could not consent	• During an epidemic some of the standard practices of research may need to be accelerated or modified in order to work in the specific context of the community and disease. For example, informed consent procedures may need to be sped up or abbreviated, or consent by proxy may be deemed appropriate in situations in which patients are not able to give consent. • Community and individual consent provided a means of making large communities aware of the research
12.	Palazzani, (2021)	Discussion	Clinical trials in the time of a pandemic: implications for informed consent	COVID-19	Clinical trial including APTs	Consent under challenging emergency contexts may require: • Re-consenting to affirm participation, for example, following changes in epidemic dynamics, or the discovery of new biomedical interventions • Waived deferred consent • Remote and internet-based consent approaches • Consent for minors, the elderly, pregnant women and ethnic minorities	• Participants should be informed of trial adaptive features (differences with RCTs) and for example that drugs deemed beneficial at the start of the trial could turn out to be harmful • The absence of validated treatments does not legitimise consent to a presumed treatment • Re-consent should accompany changes in disease dynamics, or the discovery of efficacious therapies • Deferred consent may be justified and approved by ERCS where patients may be incapable of providing consent, if have not previously objected to participation, where the risks are minimal, and where there are no alternatives. Consent may be obtained during a ‘therapeutic widow’ • While remote/telephone/internet consent approaches offer several advantages, written consent should be obtained as soon as the situation allows
13.	Papadimos *et al*., (2018)	Discussion	The ethics of clinical research during global health emergencies	General Global Health Emergencies	Trials (including adaptive/ alternative designs) in PHE	• Ebola patients could not understand the various medical interventions and so could not provide valid informed consent • Where waived or deferred, consent may be required from the next of kin • Autonomy may be compromised through fear of death - which may in fluence informed consent	• Informed consent should be prioritised during outbreaks with careful consideration for local social and cultural norms, customs, beliefs, religion, gender roles • Deferred and waived consent should be considered • Efforts should be made to understand community views on different approaches to informed consent • Important for participants to understand adaptive features of a trial
14.	Salerno *et al*., (2016)	Discussion	Emergency response in a global health crisis: epidemiology, ethics, and Ebola application	Ebola 2014–2016 West Africa	Trials (including adaptive/ alternative designs) in PHE	• Ebola context compromised participants ability to provide informed consent • The absence of treatment (lack of alternatives to research) is likely to have increased (coerced) participation and therapeutic misconception • Informed consent procedures may be waived in outbreak contexts but these do not extend to participating in clinical trials of experimental treatments in general	Balancing an autonomous informed consent process with the demand for solutions within an emergency context was highlighted as an ethical challenge
15.	Saxena, (2014)	Discussion	The ethics of the health system response to Ebola outbreak	Ebola 2014–2016 West Africa	Trials (including adaptive/ alternative designs) in PHE	Asks how informed consent can be valid in a climate of fear and how uncertainties (such as whether a trial product will be successful) can be conveyed	none
16.	Saxena and Gomes, (2016)	Discussion	Ethical challenges to responding to the Ebola epidemic	Ebola 2014–2016 West Africa	Trials (including adaptive/ alternative designs) in PHE	Community and individual informed consent required	none
17.	Woods *et al*., (2021)	Discussion	Provides a description and comparison of different methods of documenting signed consent with face- to-face and remote methods	COVID-19	NCT 04359901	Documenting written consent through: • Face-to-face • Taking a digital photo of the signed consent form • Remote, including through ‘docusign’ software	Further research needed to improve efficiency, and explore whether the requirement for documented written signed consent, rather than a witnessed oral consent, is an acceptable standard for research participants with communicable diseases
	**Empirical**						
18.	Nichol *et al*., (2021)	Empirical – qualitative study of healthcare/ humanitarian workers	Exploration of participant views on adaptive trials, engagement and consent	Ebola 2014–2016 West Africa	Trials (including adaptive/ alternative designs) in PHE	Participants felt that consent procedures needed improvement	Suggestions made towards minimising therapeutic misconceptions but none specific to APTs
19.	Cake *et al*., (2022)	Empirical – quantitative (public survey)	Survey exploring public views about trial recruitment strategies	COVID-19	PRINCIPLE evolving arms treatment trial	Online consent done for participation in online survey	None
20.	Gobat *et al*., (2019)	Empirical – quantitative (public survey)	To understand public views regarding participation in clinical research during a hypothetical influenza pandemic.	Influenza-like pandemic scenario	• Hypothetical APTs • Therapeutics – primary care and intensive care scenarios • Response adaptive randomisation	• For primary care studies 3972 (58.4%) participants preferred prospective written informed consent, 2327 (34.2%) thought simplified procedures would be acceptable. • For ICU studies, 2800 (41.2%) preferred deferred consent, and 2623 (38.6%) preferred prospective third-party consent. • Support was expressed for the use of routine medical data for pandemic research without explicit consent • Simplified consent forms would be acceptable in pandemics though a lack of understanding could result in therapeutic misconceptions • Adaptive trials may be conceptually hard to understand for participants, but respondents prioritised information about trial risks over details about study design	• Study indicated public support for clinical research in pandemics • Tailored information and initiatives to advance research literacy and maintain trust are needed to support engagement and research participation during pandemics
21.	Gobat *et al*., (2018)	Empirical –qualitative - focus groups and interviews	To identify public views regarding provision of information and consent to participate in primary and critical care clinical research during a future influenza- like pandemic (Belgium, Spain, Poland and the UK)	Influenza-like pandemic scenario	• Hypothetical APTs • Therapeutics – primary care and intensive care scenarios • Response adaptive randomisation	• Ethically robust research procedures were appreciated by participants • Lengthy enrolment/consent procedures were seen as a barrier to recruitment • They proposed simplified enrolment processes for higher risk research and consent waiver for certain types of low-risk research • They supported using routinely collected, anonymized clinical biological samples for research without explicit consent for non-commercial purposes	More proportionate research protection procedures were recommended for publicly funded, low-risk research, to facilitate rapid recruitment
	**Trial reports**						
22.	Ali Karim *et al*., (2022)	Publication – Trial report	COVID-19 Trial results - treatment of in hospitalised patients	COVID-19	COVID-19 (CATCO)	Consent was either obtained a priori or deferred, as per the requirements of local ethics boards.	None
23.	Angus *et al*. (2020)	Publication – Trial report	Effect of Hydrocortisone on Mortality and Organ Support in Patients with Severe COVID-19	COVID-19	REMAP-CAP	Written or verbal consent, in accordance with local legislation, was obtained for all patients or from their surrogates.	None
24.	Henao- Restrepo *et al*., (2017)	Publication – Trial report	Final results from the Guinea ring vaccination, open-label, cluster-randomised trial (Ebola Ça Suffit!)	Ebola 2014–2016 West Africa (Guinea and Sierra Leone)	Ebola Ca Suffit! ring vaccination trial	• Written informed consent from all eligible contacts using a printed information sheet. • For illiterate participants ICFs were read in the local language and a fingerprint was taken in place of a signature • Eligible contacts and were informed of the outcome of the randomisation at the end of the informed consent process to avoid selection bias	None
25.	Horby *et al*., (2020)	Publication – Trial report	RECOVERY trial results	COVID-19	RECOVERY	Written informed consent was obtained from all patients or from a legal representative if participants were too sick	None

### Ebola 2014-2016 ADTs

Included articles provided rich descriptions of informed consent challenges and approaches ^39, 41, 45– 47, 50, 51^. A common challenge reported for RCTs in general and also for therapeutic and vaccine ADTs was the complexity of research concepts and procedures and difficulties communicating these, including among participants with low literacy ^3, 39, 48, 50^. Vulnerability, comprehension of trial concepts and therapeutic misconceptions (a phenomenon where research participation is motivated by individual participants' overestimation of the therapeutic or protective properties of experimental interventions ^68^) were described as challenges to autonomous, informed consent. Fostering culturally sensitive consent processes and an understanding of trial procedures through consultation with the community was recommended ^59^. Lengthy information sheets and informed consent forms may also present a barrier to full understanding of trial processes and to trial enrolment ^3, 5, 60, 61, 69– 71^. The context of public health emergencies, where disruption, fear and confusion are common, was also described as presenting challenges for informed consent ^41, 45, 51^ and potential for therapeutic misconceptions ^45, 51^. Alirol and colleagues described this as ‘situational coercion’ ^48^, and whilst this may result in increased trial participation, it could potentially lead to long-term public mistrust in research ^61^. For some trials, because of the PHE context, ethics review committees granted consent waivers ^45, 51^ and community members who wished to decline research participation could self-identify through wearing bracelets ^45^. Where informed consent was required by a community leader and the individual participant, trial staff used a combination of storyboards and flipcharts ^39, 50^ in group and individual meetings in an attempt to convey trial information to potential trial participants ^39, 46, 47^. Trial participation also raised community hopes of protection from vaccination, and for ring vaccination trials where community members desired immediate in preference to the delayed vaccination, randomisation into immediate or delayed vaccination was done prior to consenting to avoid selection bias ^46^.

### COVID-19 APTs

COVID-19 trial reports provided short descriptions of their informed consent approaches ^17, 72, 73^: For the REMAP-CAP trial, written or verbal consent was obtained for all patients or from their surrogates ^17^; for the CATCO trial, consent was obtained a priori or deferred ^72^; and for the RECOVERY trial, written informed consent was obtained from all patients or from a legal representative if they were too sick ^73^.

A key issue for informed consent to APTs relates to the information that is provided regarding trial-specific adaptations and the optimal level of detail about trial design. In a PHE, streamlined consent processes and simplified consent forms are important for rapid enrolment ^70, 71, 74, 75^. Potential challenges to simplified informed consent processes are unfamiliarity of research stakeholders with APTs ^62^ and the possibility of therapeutic misconceptions, argued to be heightened in PHE contexts ^3, 60, 61, 76^. Some articles call for clarity in informed consent forms and processes as a means of mitigating against therapeutic misconception ^59, 69, 77^ and Palzzani, in her ethical discourse, highlights the importance of giving complete, non-paternalistic information and verifying the participant’s understanding ^69^ *.* For APTs, Palazzani recommends a dynamic consent process emphasising that participants should be informed about; the trial design, its adaptive nature and the possible addition and removal of trial arms based on efficacy and futility respectively, how it differs from traditional trials, and implications for participation ^69^. Further, she recommends that re-consenting should inform participants of any new beneficial treatment uncovered since the initial consent was conducted ^69^. Formative research for clinical research in PHE from high income countries highlighted that publics prioritised information on the implications of adaptive designs on their participation, including on burdens, risks and benefits of research participation, over theoretical explanations of the study design ^61^.

Of Informed consent information sheets and assent forms (for minors) reviewed for 6 large COVID-19 APTs ( Table 3), five provided information to patients that they would be randomised into one of several therapeutic arms, giving descriptions about potential adverse effects for each trial therapy ^15, 16, 18, 56, 64^. One of the information sheets stated that participants would be notified if trial arms were ‘interrupted’ ^16^ and another stated that *“The study looks at its results as it goes and uses the results so that new patients in the study have a better chance of getting better treatments”* (Assent Information form acquired from the REMAP-CAP ^17^ research team). Of the remaining 5 APT information sheets, none provided information that new trial arms could be added. For the COVID-19 (TICO/ACTIV-3), Murray *et al*., ^74^ describe the use of "modular consent forms" with separate appendices for each trial arm giving detailed information to participants about the intervention they were randomised to. This presumably negated the need to give detailed information to participants on all trial interventions, thus shortening the informed consent form. This approach also eased the process of adding new trial arms, given that details for participants were added to the ICF and protocol as an appended module. REMAP-CAP described the adaptive nature of the trial in their public facing website.

In PHEs there is a need to minimise and mitigate against infection during the informed consent process. Mitigating strategies, such as social distancing, isolation, and the need for research staff to wear PPE were described as barriers to communication during the consenting process for Ebola ^48^ as well as COVID-19 trials ^69, 70, 75^. For COVID-19 APTs, face-to-face approaches were used for some studies as well as other innovative remote approaches ^16, 17, 66, 67^. Remote consent approaches, though logistically challenging to implement ^69^, have been recommended to minimise infection ^69, 70^, and with ethical approval, trials have embraced this approach both online ^15, 18, 67^ and by phone ^15, 17, 64^. For REMAP-COVID, after screening, patients were introduced to the trial in a one-on-one 'teleconferencing' call with a trial staff member ^56^. Declaration of informed consent was then facilitated through a software platform facilitating signing and countersigning. Face-to-face support was also provided for consenting where it was needed and the team anecdotally report that remote consent was most effective when combined with bedside engagement, though they provide no detail of how this was assessed ^56^. Questions were raised in the literature about informed consent being administered by ‘treating’ clinicians with limited consent training (as opposed to trained research clinicians) in ‘pragmatic’ trials such as RECOVERY ^20^ and about the validity of online and remote approaches in confirming with certainty, the identities of consent signatories ^78^. While RECOVERY provided mandatory online training for clinicians, Casey *et al*., suggest that in ‘pragmatic adaptive platform trials’ consent processes could be supported by community consultation and information ^20^. Woods *et al*., provide a detailed description and comparison of different approaches of obtaining written consent whilst minimising the risk of infection through face-to-face, taking a photo of the form and using Docusign software ^75^. The review has highlighted several innovations in modifications of consent processes to address PHE challenges, but no study described systematic methods to explore their effectiveness and / or the specific challenges related to APTs in this context.

Optimising recruitment and informed consent processes to enable rapid recruitment is a common theme to discussion articles on COVID-19 and on GHEs in general particularly for low risk studies involving repurposed drugs with good safety profiles ^60, 61, 70, 71, 78^. For incapacitated patients, deferred consent, or consent by proxy by family members or legal representatives has been recommended and approved by ethics committees, with a further recommendation that patients can later be consented during therapeutic windows ^5, 75^. Monach *et al*., describing the range in trial candidates, from RCTs exploring different doses of drugs with known safety profiles and off-label drugs with a well-described safety profile, to investigational drugs with unknown safety profiles, recommends a nuanced approach to consent ^71^. For low risk trial candidates, waivers (opt-out) they argue, can be considered, whilst opt-in oral and written consent is more appropriate for higher risk trial candidates with unknown safety profiles. Specific issues as they relate to APTs were not considered in these articles.

## Discussion

In this review of community engagement and informed consent for ADTs and APTs in PHEs, we found that current guidance documents place strong emphasis on the importance of community involvement in conducting trials, but little specific guidance on what this means in practice for ADTs and APTs. Based on reported experiences of conducting clinical trials in public health emergency contexts, important lessons have been learned regarding how community engagement and informed consent processes can be optimised. There is a gap in consistent reporting of these experiences and lessons, in particular as they relate to the novel aspects of trial design and the implication for participants. Based on this review, we highlight key considerations for best practice in community engagement and informed consent relevant to ADTs and APTs for PHEs which may helpfully be included in future guidance.

### Key considerations for engagement In ADTs/APTs in PHEs

Pre-trial engagement is important for gaining understanding of community norms, values and traditions which might have a bearing on the way in which a trial may be implemented, assessing trial acceptability and feasibility by the community, and drawing community insights to inform design selection ^3, 23, 27, 32^. This demonstrates respect for populations, aims at nurturing trust in research and acknowledges that the perception of social value of research can vary between local stakeholders and research stakeholders, particularly in international research collaborations ^79^. The review has identified a range of approaches that reflect good practice for GPP/ engagement in alternative design trials for Ebola ^34, 45, 46, 50, 52, 80^ and also for multi-site, multi-country COVID-19 trials delivered according to a master trial protocol ^6, 42, 43, 55^. However, we found limited description of pre-trial engagement that could meaningfully impact study design or implementation. The exception was qualitative work that considered public views of APTs and consent for pandemic relevant research that was conducted for PREPARE, a clinical research preparedness infrastructure ^60, 61^ and APT protocols that explicitly mentioned use of CABs and PPI panels (Re: PRINCIPLE). In a public health emergency the time to engage publics in design aspects is exceptionally tight. To enable pre-trial engagement in these contexts, strong engagement and communications practices need to be built into the infrastructure for these research platforms. Further, ground work for building CABs and public engagement links should be established between emergency events for rapid activation during readiness for research response. This work can and should include building a suite of public-facing communications tools that can be adapted to explain different aspects of ADTs or APTs relevant to PHEs. Capacity development materials for a wide range of stakeholders, including CABs or PPI groups would also be of value.

GPP-EP guidelines emphasise the importance of public acceptability of trials ^2, 3, 23, 32, 35^. The dynamic nature of APTs delivered in PHEs mean that engagement regarding acceptability and social value should continue throughout the trial’s lifetime. First, adaptive trials, by design, aim at validating therapies/vaccines throughout their implementation. Thus, if they are successful, as they proceed new alternatives for addressing the health problem emerge. That social value, “a necessary component of acceptability” ^79^, is related to the availability of alternatives to address the problem, and implies that as validated alternative therapies/vaccines emerge, the social value of the trial changes correspondingly. Secondly, given the longer timescales of adaptive trials (for example, RECOVERY celebrated its third anniversary in March 2023), the emergence of efficacious vaccines and dynamic biological factors including virulence, endemicity and heard immunity change over the trial’s lifetime, and therefore, correspondingly, so does the disease’s impact on populations. So, while continuous engagement throughout conventional RCTs may be important to re-assess acceptability and social value as new therapies may emerge and the disease evolves, arguably this is more important for adaptive trials because of their longer timeframes within a dynamic epidemic context, and because continuous identification of new therapies and vaccines as trials progress is at the heart of their design. Considerations for guidelines to inform engagement during the implementation of adaptive platform trials might include: how to engage communities when an arm is stopped for futility or efficacy; engaging communities for the introduction of new arms; and re-assessing social value as validated therapies/vaccines emerge.

While several guidance documents underscore the importance of planning respectful exit strategies through consultation ^2, 33, 35^ and appropriate ways of sharing trial results, in order to maintain trust and respectful ongoing partnerships ^2, 3, 23, 27, 33, 35^, the review yielded no descriptions of how this was done for any adaptive or alternative trial.

### Key considerations for informed consent in ADTs and APTs in PHEs

We highlight three important questions about informed consent forms and processes for ADTs/APTs in PHE contexts. Firstly, given the greater complexity of ADTs/APTs ^62, 69^, what level of detail on the adaptive/alternative nature of trials should informed consent forms convey; secondly, how effective are remote and online processes in achieving informed consent and ensuring the validity of participant identity; and lastly, for pragmatic APTs, how can modified consent processes be improved and ‘treating’ clinicians be supported further to administer informed consent which minimise therapeutic misconceptions ^68^? Palazzani emphasises that participants should be informed about the adaptive nature of the trial ^69^ and research among European publics highlighted their preference to understand the implications of the adaptive nature of the design for them regarding the risks, burdens and benefits ^61^. Balancing the need to avoid PHE contexts precipitating research exceptionalism through enabling reduced quality processes, including in informed consent ^81^, with the need for the rapid ethical evaluation of therapies and vaccines, requires careful navigation. Casey and colleagues argue that tight regulation would have prevented the implementation of RECOVERY in the USA, but that the protocolisation of research integrated into care in the UK minimised the wide clinical use of unproven therapies like hydroxychloroquine ^20^. On one hand, over-complicated information materials, consent forms and processes can impede research ^60, 61, 70, 71^, while therapeutic misconception, on the other, can lead to long-term mistrust in research ^61^.

In the context of placebo controlled COVID-19 vaccine APTs, Singh and colleagues’ guidelines specify that when authorized vaccines become available, participants should be offered an opportunity for unblinding and vaccination with the authorized vaccine or the investigational vaccine if proven to be efficacious ^34^. However, the review revealed no empirical articles on consent for COVID-19 adaptive vaccine trials. Questions of relevance to these trials pertain to participation in trials during national roll-out of approved vaccines, and include: a) how have (or should) consent forms conveyed potential restrictions to participant freedom, for example, to travel or work, when they opt for vaccine trial participation instead of nationally provided vaccines; b) how do consent processes address the tension between encouraging uptake of nationally provided vaccines and recruiting trial participants; and c) how should consent processes and ICF content change with the evolving public health regulations in different countries.

### Strengthening practice and evidence for what works

We identified some examples of good practice based on published reports and personal communication with those leading prominent APTs during COVID-19. However, there were many other platform trials delivered during the pandemic - one review identified 58 COVID-19 platform trials by May 2021 ^30^. Given this large number, the scarcity of engagement reports in the literature is surprising. There is a need for norms and guidance related to minimum standards for reporting of GPP-EP practices. In particular, reporting ways in which trial adaptations were communicated with trial participants and at which junctures would help to inform best practice in this work. Addressing this gap would further enable researchers to be accountable to their peers and broader publics in describing how public acceptability of research was determined; how public views were taken into account; how public input was incorporated into trial design and implementation; and if some views could not be incorporated, what were the reasons for the decisions taken.

Our review revealed novel approaches to informed consent aimed at broadening participation whilst minimising transmission, but no empirical studies explored what content informed consent forms adaptive platform trials should contain, nor the effectiveness of the novel delivery and consent training approaches. There was also no empirical evidence on the effectiveness of online or remote consent approaches and support materials (leaflets, animations etc). Further research is needed aimed at strengthening these areas. Given the significant challenges of conducting research on research, particularly during emergency events, we recommend that pragmatic, rapid operational approaches to evaluation are adopted and included in trial protocols. This planning is needed well in advance of emergency events.

### Strengths and limitations

To our knowledge, this is the first review that considers the practice of community engagement and informed consent for novel trials conducted during PHEs. This review is timely given the rich learning that has happened through unprecedented investment in and delivery of clinical research during COVID-19. Further learning could have been gained by expanding our review to include oncology and critical illness clinical research, where adaptive trials and relevant aspects such as consent waiver/ deferral are more commonly encountered. We maintained our focus on PHE contexts, however, due to their specific features, including the urgency for rapid research responses and the unavailability of existing known medical countermeasures during these events. Our search was comprehensive and covered peer-reviewed and grey literature. We also drew on consultations and information for COVID-19 trials. Further information to inform key outcomes to this review may have been gathered through a greater focus on grey literature and talking to research teams. Given time constraints to conduct this work, we selected a rapid review methodology. One member of the research team identified included citations and extracted data, with 10% of the data double coded. This may have introduced error in the research process. Further, limiting our inclusions to English language papers only may have introduced bias in reporting.

## Conclusion

COVID-19 witnessed a step change in the delivery of clinical research during PHEs. These advances led to identification of effective medical countermeasures to save lives during the pandemic. Prominent clinical trials such as RECOVERY, SOLIDARITY and REMAP-CAP received widespread media coverage and investigators and their teams played significant and active roles in engaging the public with research. These breakthroughs demonstrate the added value of novel trial designs that enable rapid and responsive clinical research integrated into health emergency response. It is vital that lessons learned from these advances inform guidance for rapid research in future events, including for strong and consistent best practice for community engagement and informed consent.

In our review, we identified a gap in current guidance documents to steer best practice for community engagement and informed consent for ADT and APTs during PHEs. We also found some description and evaluation of practice that can inform future guidance. To advance best practice and be accountable to collaborators and communities, trial teams must report on their engagement and informed consent practices for ADTs and APT in PHEs.

## Data Availability

All data underlying the results are available as part of the article. OSF: A rapid review of community engagement and informed consent processes for adaptive platform trials and alternative design trials for public health emergencies https://doi.org/10.17605/OSF.IO/YH6AB ^29^ This project contains the following extended data: 20230413_wellcomeopenres_Supplementary Material.docx (search strategies) OSF: PRISMA checklist and flowchart for ‘A rapid review of community engagement and informed consent processes for adaptive platform trials and alternative design trials for public health emergencies’. https://doi.org/10.17605/OSF.IO/YH6AB ^29^ Data are available under the terms of the Creative Commons Attribution 4.0 International license (CC-BY 4.0).
