# Supplementary material for: A rapid review of community engagement and informed consent processes for adaptive platform trials and alternative design trials for public health emergencies
Source: Wellcome Open Res. 2023 May 2;8:194. [Version 1] doi: 10.12688/wellcomeopenres.19318.1 (PMC10465998; doi:10.12688/wellcomeopenres.19318.1)
Supplement: Peer review history

# Peer review: Reviewer response for version 1

**DOI:** 10.21956/wellcomeopenres.21401.r65428

**Published:** 2023-08-29

**Authors:** Byron Bitanihirwe

**Affiliations:** 1 The University of Manchester, Manchester, England, UK

This is a compelling review focused on identifying existing guidance, reports and evidence related to informed consent and community engagement for alternative design trials (ADTs) and adaptive platform trials (APTs) during public health emergencies (PHEs), mainly in relation to COVID-19 and the Western Africa Ebola epidemic. The authors conducted a meticulous and comprehensive interrogation of 8 electronic databases as well as grey literature to answer this question. Based on the 49 articles that met inclusion criteria, the authors found that while current PHE clinical research guidance documents appreciate the significance of ADTs and APTs in addition to the importance of community involvement in conducting trials, there unfortunately remains a lack of specific guidance as to what this means in practice for both these forms of trials. Another key finding from the rapid review involved the existing gap in reporting of experiences associated with conducting clinical trials during PHEs and how these experiences relate to novel aspects of trial design, particularly in terms of community engagement and informed consent processes. It follows that each of these findings are crucial especially as one considers aspects of best practice for optimising large-scale testing of interventions during PHEs based on lessons learned from previous trials.

 Overall, this is a well conducted and timely study (with clearly identified limitations) that draws attention to a pressing public health challenge, especially as emerging pandemics/epidemics spread faster and further.

Are the rationale for, and objectives of, the Systematic Review clearly stated?

Yes

Is the statistical analysis and its interpretation appropriate?

Not applicable

Are sufficient details of the methods and analysis provided to allow replication by others?

Yes

Are the conclusions drawn adequately supported by the results presented in the review?

Yes

Reviewer Expertise:

Global Health and Translational Psychiatry

I confirm that I have read this submission and believe that I have an appropriate level of expertise to confirm that it is of an acceptable scientific standard.

# Peer review: Reviewer response for version 1

**DOI:** 10.21956/wellcomeopenres.21401.r61454

**Published:** 2023-07-14

**Authors:** Monica Schoch-Spana

**Affiliations:** 1 Department of Environmental Health and Engineering, Johns Hopkins Bloomberg School of Public Health, Baltimore, MD, USA

At the behest of the WHO Ethics and Governance Unit and to inform potential practice improvements, the authors systematically reviewed literature concerning community engagement and informed consent in relation to Alternative Design Trials (ADTs) and Adaptive Platforms Trials (APTs) - clinical research approaches intended to generate evidence in support of appropriate responses to public health emergencies (PHEs) when time is of the essence.

 The authors have made clear their purpose: to identify what is known about engagement and informed consent practice in ADT/APT contexts and how clinical trials might improve going forward. They have also outlined in sufficient detail - that would allow for replicability - their methods for conducting the literature review.

 Three major, well supported observations emerge from the literature review:

- First, whereas prominent guidance documents argue for the importance of incorporating community engagement and informed consent for ADTs and APTs during PHEs, these reports provide limited instruction in best practice.
- Second, as input for filling this gap, recent clinical trials during PHEs - including COVID-19 and 2014-2016 Ebola - reveal a range of challenges and opportunities that emerge when investigators involve the people and communities directly affected by the research as collaborators rather than as mere study subjects.
- Lastly, the ad hoc nature in which researchers tend to report on engagement and informed consent processes suggests the need for greater documentation, transparency, and shared benchmarks regarding the social/relational aspects to ADTs and APTs, on par with the more technical/analytical dimensions.

The authors have provided information on engagement and informed consent processes at such a granular level that it would be of immediate benefit to individuals planning ADTs/APTs should a PHE unfold tomorrow (and more universal guidance is not yet available), and they have successfully catalogued concrete considerations with which clinical researchers and their partners could begin developing, over a longer term, more comprehensive guidance on best practices going forward.

Are the rationale for, and objectives of, the Systematic Review clearly stated?

Yes

Is the statistical analysis and its interpretation appropriate?

Not applicable

Are sufficient details of the methods and analysis provided to allow replication by others?

Yes

Are the conclusions drawn adequately supported by the results presented in the review?

Yes

Reviewer Expertise:

A medical anthropologist, I have worked as a researcher and policy analyst/advisor in the field of public health emergency management for over 20 years, with a focus on community resilience, community engagement, whole community approaches to disaster management, behaviorally realistic emergency planning, and crisis and emergency risk communication.

I confirm that I have read this submission and believe that I have an appropriate level of expertise to confirm that it is of an acceptable scientific standard.
